# Supplementary material for: Association of depression and antidepressant therapy with antiretroviral therapy adherence and health-related quality of life in men who have sex with men
Source: PLoS One. 2022 Feb 25;17(2):e0264503. doi: 10.1371/journal.pone.0264503 (PMC8880848; doi:10.1371/journal.pone.0264503)
Supplement: S1 Table — (DOCX) [file pone.0264503.s001.docx]

| **Table S1. Medication Adherence Report Scale (MARS-5).** | | |
| --- | --- | --- |
| **Item number** | **Question** | **Response** |
| MARS-Q1 | I forget to take my anti-retroviral drugs | □ (1) Always  □ (2) Often  □ (3) Sometimes □ (4) Rarely  □ (5) Never |
| MARS-Q2 | I alter the dose of my anti-retroviral drugs | □ (1) Always  □ (2) Often  □ (3) Sometimes □ (4) Rarely  □ (5) Never |
| MARS-Q3 | I stop taking my anti-retroviral drugs for a while | □ (1) Always  □ (2) Often  □ (3) Sometimes □ (4) Rarely  □ (5) Never |
| MARS-Q4 | I decide to skip a dose of my anti-retroviral drugs | □ (1) Always  □ (2) Often  □ (3) Sometimes □ (4) Rarely  □ (5) Never |
| MARS-Q5 | I take less anti-retroviral drugs than instructed | □ (1) Always  □ (2) Often  □ (3) Sometimes □ (4) Rarely  □ (5) Never |
