# Supplementary material for: Association of depression and antidepressant therapy with antiretroviral therapy adherence and health-related quality of life in men who have sex with men
Source: PLoS One. 2022 Feb 25;17(2):e0264503. doi: 10.1371/journal.pone.0264503 (PMC8880848; doi:10.1371/journal.pone.0264503)
Supplement: Attachment — Submitted filename: Review for Plos One_Yen et al_Depression among MSM on ART_Taiwan_17 May 2021_ib.docx [file pone.0264503.s003.docx]

Yen et al: **Association of Depression and Antidepressant Therapy with Antiretroviral Therapy Adherence and Health-Related Quality of Life in Men Who Have Sex with Men_Taiwan**

Review for Plos One, 17 May 2021

This manuscript adds to scientific knowledge and data on the important issue of mental health and specifically depression among HV-positive MSM on ART. The study draws attention to the need for greater attention to the systematic integration of mental health care as part of comprehensive HIV and health services for MSM.

Broader comments:

- There are two issues that should be acknowledged as limitations to this study:
  a) Participants enrolled include only MSM already enrolled in HIV and health care services, thus not capturing mental health issues or depression among MSM not accessing health facilities. A recommendation for further study of depression could be made that facility-based data should be complemented by data form a survey such as an MSM IBBS that would collect information also from those not accessing facility-based services. It is possible that rates of depression might be higher or even higher among those not accessing services.
  b) The scope and/or funding for this study may not have allowed for this, it would however have been best to collect and test biological samples for ARVs to assess ART adherence rather than to rely on self-reported behaviors.
- The authors make np mention f involvement of MSM or MSM CSOs in the design, implementation or monitoring of this study. If this has been done, it should be added. If it has not been done, it is an important submission and should be strongly recommended for any future studies.

Detailed comments:

- Line 77-78: This would be true in the absence of other prevention measures being used, e.g. correct and consistent condom use, HIV-negative partner(s) on PrEP.
- Line 83-84: Somewhat misquotes the new UNAIDS targets. This should correctly quote the new UNAIDS 2025, with the only relating or covering this use as “90% of People Living with HIV and People at Risk are Linked to Other Integrated Health Services”, which mental health services would be part of. (Source: “End Inequalities. End AIDS. Global AIDS Strategy 2021-2026”, UNAIDS 2021)
- Line 113-114: Sentence is unclear and suggest that enrolment has not been limited to MSM but included other PLWHA. While this is clarified in the method section later (stating only MS were eligible), it should be clarified or corrected here also.
- Line 136-137: Appreciate the adaptation and addition of items specific to Taiwanese culture. Could you kindly provide some background to the item “eating what one likes to eat” to the environmental domain, given many readers may not be familiar with the Taiwanese culture?
- Line 194-196: Please add AOR, CI and p-value for these associations, i.e. lower income, drug use, gonorrhea.
- Line 238-239: Date are provided on depression from the US, SSA and France. What are baseline data or estimates for Taiwan or for the Asia region as more relevant (if known)?
- Line 276: First sentence in the conclusion section should specify or add that this applies for clinical sites in Taiwan, where MSM were accessing services.
