# Supplementary material for: Association of depression and antidepressant therapy with antiretroviral therapy adherence and health-related quality of life in men who have sex with men
Source: PLoS One. 2022 Feb 25;17(2):e0264503. doi: 10.1371/journal.pone.0264503 (PMC8880848; doi:10.1371/journal.pone.0264503)
Supplement: Attachment — Submitted filename: PONE-D-21-07210 Reviewer comments.pdf [file pone.0264503.s004.pdf]

Title: Association of Depression and Antidepressant Therapy with Antiretroviral Therapy Adherence and Health-Related Quality of Life in Men Who Have Sex with Men

Overview and general comments: Authors describe the association between depression/antidepressant therapy and adherence and health related quality of life (HRQOL) among MSM enrolled at hospital HIV clinics in Taipei, Taiwan. The authors found a positive association between presence of depressive symptoms and poor adherence and HRQOL. The manuscript is well written, and the authors work addresses a topic of critical importance to achieving viral suppression and ending the HIV epidemic.

Specific comments:

Abstract: Very clear and concise..

Introduction: The authors might want to correct the interpretation of the 2nd in UNAIDS 90-90-90 to "sustained ART" instead of started ART (line 72-73)..

Method: Methods address the study goals and as a strength, the authors added some culturally relevant questions to the HRQOL measures. The authors do not describe the total number of MSM in the clinics and how the samples size was arrived at or was adequate.

Results: 565 MSM enrolled but not clear if this was a representative sample for MSM in the clinic. Was this an adequate sample? See comment in the methods section.

Line 194. Small typo. Use "terms" instead of "term"

Line 200. After controlling "for" instead of controlling the

Line 221. Use controlling "for"

Conclusion: Results should not be repeated in the conclusion verbatim. Line 278-281 should be rephrased since it appears in the results section almost in the same way.
